# Short‐term inhibition of SARS‐CoV‐2 by hydrogen peroxide in persistent nasopharyngeal carriers

**DOI:** 10.1002/jmv.26485

**Published:** 2020-09-24

**Authors:** Amedeo F. Capetti, Fabio Borgonovo, Valentina Morena, Angelica Lupo, Maria Vittoria Cossu, Matteo Passerini, Gianfranco Dedivitiis, Giuliano Rizzardini

**Affiliations:** ^1^ Department of Infectious Diseases “Luigi Sacco” University Hospital Milano Italy

**Keywords:** COVID‐19, hydrogen peroxide, nasopharingeal, persistent, SARS‐CoV‐2, swab

## Abstract

Asymptomatic and convalescent coronavirus disease 2019 (COVID‐19) subjects may carry severe acute respiratory syndrome coronavirus 2 (SARS‐CoV‐2) for months in their upper respiratory ways. Desiring to permanently clean the mucosal surfaces, we investigated the chemical agents that fit to rapidly degrade the virus. Among these, hydrogen peroxide, initially tested by two of us for tolerability, showed both good performance and acceptable side effects (burning sensation for 15–20 s). We contacted circles of family physicians and the ATS Milano (Territorial Assistance and Prevention Service), and we tested this procedure on eight persistent carriers of SARS‐CoV‐2, performing swabs before the procedure and after it until the reappearance of the virus or until 14 days (the incubation period), keeping the surfaces clean with a hypertonic solution. Our patients had a median time from exposure or symptom onset of 111 days, and three had relapsed after being declared “cured” (two consecutive negative swabs after quarantine). One patient had a baseline negative swab and was excluded, and two successfully ended the 14 days' course, four suppressed viral elimination for 72 h, and one for 48 h, all rebounding to weak positive (cycle thresholds above 24). Although temporarily effective, such measures may have some place in the control of viral shedding to protect the most fragile subjects.

## 1 INTRODUCTION

Asymptomatic subjects as well as convalescents, even in the presence of antibody response, may remain nasopharyngeal carriers of severe acute respiratory syndrome coronavirus 2 (SARS‐CoV‐2) for a long time.^1^, ^2^ The issue to what extent these carriers may contribute to the spread of coronavirus disease 2019 (COVID‐19) is still debated.^3^, ^4^ As time since disease onset appears to be linked to higher cycle thresholds (*C*_t_) in positive PCR results and to lower contagiousness.^5^ The US Centres for Disease Control and Prevention and the WHO have introduced time‐lapse criteria to end quarantine, avoiding the repetition of swabs.^6^, ^7^ The Italian Ministry of Health, however, still adopts quarantine measures for all those subjects whose swab has proven positive and for their contacts.^8^

Among the chemical agents able to kill SARS‐CoV‐2, hydrogen peroxide (H_2_O_2_) 3% is well‐studied,^9^, ^10^ kills the virus in 30 s, and is best for mucosal cleaning.^11^ It also promotes destruction of RNA in 5 min through the activation of free radicals.^12^

In this study, we describe our attempt to reduce the presence of SARS‐CoV‐2 in the nose and throat of eight long‐term carriers by washing their mucosa with a hypertonic solution and subsequently with H_2_O_2_ 3%. This process has been suggested by some colleagues, but to our knowledge, no data exist to date about its efficacy.^13^

## 2 MATERIALS AND METHODS

### 2.1 Patients inclusion criteria

Patients could be included if aged over 18 years, with documented SARS‐CoV‐2 infection with either continued positive swabs for more than 60 days or reappearance of positive swabs after the declared end of the disease (two consecutive negative swabs at the end of the quarantine period), last swab result: positive (less than 24*C*_t_), without nasopharyngeal malformation or swallowing alterations, able to understand and provide written informed consent.

### 2.2 Patients source

Groups of family physicians and the Territorial Assistance and Prevention Service (ATS) of Milan were informed of our procedure and offered this possibility for long‐term carriers of SARS‐CoV‐2.

### 2.3 Procedure and laboratory test

Nasopharyngeal swabs were performed by brushing the pharynx through the oral cavity and the nasopharynx through both nasal choanae or only the open one in case of severe obstruction (i.e., septal deviation, polyps).^14^, ^15^ The PCR commercial test used three pairs of primers designed to target RdRp, E, and N genes. The detection of these genes within 24*C*_t_ is reported as positive, above 24*C*_t_ as weak positive, and no amplification within 40*C*_t_ was considered negative. Neutralizing antibodies, directed against epitopes in the S1/S2 region of the virus, were detected through the LIAISON™ kit by DiaSorin.

### 2.4 Nasopharyngeal washing and swab schedule

A baseline nasopharyngeal swab was performed to check that the patient was still a carrier of SARS‐CoV‐2. Subsequently, patients were provided with Atomix® Wave™ kit for nasopharyngeal washing, filled the micropump, and cleaned both choanae once, bending forward.^16^ The procedure was repeated using pure H_2_O_2_ 3% solution and patients were asked to wash their mouth and perform 2ʹ gargles and then spit, avoiding to swallow. Daily nasal cleaning with Atomix® Wave™ was indicated for 14 days. The patients repeated swabs at 24, 48, and 72 h, and, if still negative, at 7 and 14 days.

### 2.5 Ethical aspects

The procedures were performed in compliance with relevant laws and institutional guidelines and in accordance with the ethical standards of the Declaration of Helsinki.

The Luigi Sacco Hospital Ethics Committee was informed, but since no Investigational New Drug was involved, specific approval was deemed unnecessary.

Written informed consent was obtained before any procedure was performed.

## 3 RESULTS

### 3.1 Patients' clinical history

Seven patients up to date have been included in this procedure. One dropped out early due to a negative baseline swab. Patients' median age was 38 years (range, 29–54), two were male and two had underlying immune depression (cancer and sarcoidosis). The median antibody production was 21.05 UA/ml (range, 12.2–57.3), overall quite low but invariably positive.

**Figure 1:**
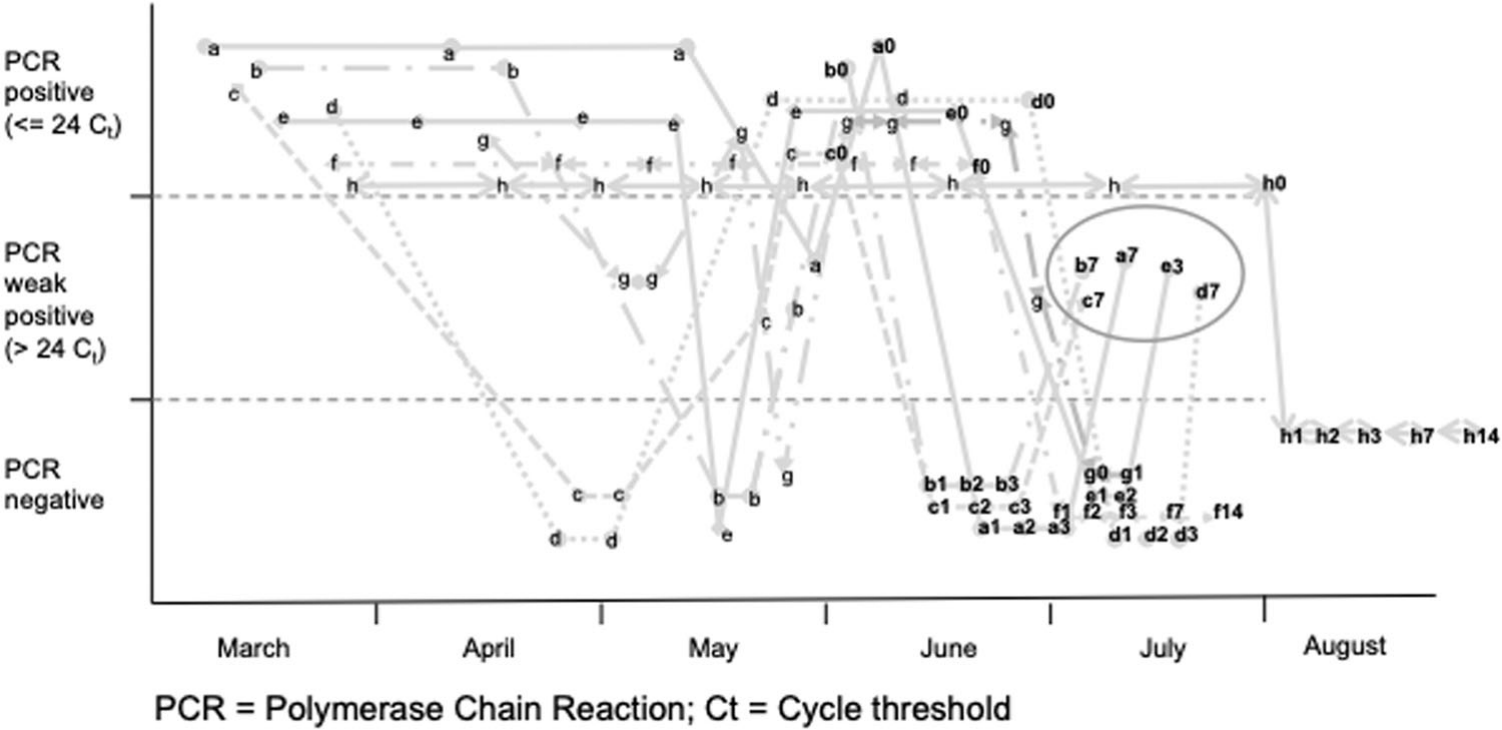
Time course of swabs in the eight patients. Each patient is identified by a letter. Letters followed by numbers indicate their relationship to hydrogen peroxide intervention (ie: a0 indicates just before the intervention, a3 three days later, etc). The five rebounds are circled. *C*_t_, cycle threshold; PCR, polymerase chain reaction

The median time from symptom onset to the intervention was 111 days (range, 103–130) and the median number of positive swabs before the intervention was 4 (range, 3–8). Three patients had in their past one weak positive PCR and one had two, while five patients had at least one negative PCR, three being temporarily declared “cured” (see Figure 1).

### 3.2 Patients' outcomes

One patient had a negative swab at baseline and was therefore excluded from follow‐up. Two patients had prolonged negative swabs until Day 14 and was declared “cured.” One patient had negative swabs until 48 h but returned weak positive at 72 h.

Four patients had negative swabs until 72 h but returned weak positive at day 7. The median time to rebound was therefore 168 h (range, 72–168).

None returned to positive PCR. No correlation was observed with antibody levels, nor with the time from symptom onset, which was quite homogeneous, nor with the number of nonpositive or negative PCR results in their history.

## 4 DISCUSSION

The present case series, though small, confirms suspicions on the course of COVID‐19, which cast a new, worrying light on it. First of all, nasopharyngeal carriers can persist for up to 128 days, which is much longer than previously reported.^17^ Second, they can coexist with low‐level neutralizing antibody production, which suggests that subjects with low antibodies may deserve special attention. Third, even patients declared “cured” might return to a productive status, and maybe they never got rid of the virus, as we have seen in COVID‐19 pneumonia patients who had repeatedly negative swabs before diagnosis.^18^ The fact that even washing with H_2_O_2_ may cause only temporary interruption in viral shedding suggests that the virus may continue to replicate either in deeper mucosal strata or in the bronchial epithelium. In any case, it seems desirable to lower the local production of viral particles and viral RNA, although temporarily. We are planning to propose more intense nasopharyngeal washing with H_2_O_2_ in a 14‐day period (epithelial turnover time) to verify if this may cause more profound suppression of SARS‐CoV‐2. In any case, we believe that, since H_2_O_2_ has proven to suppress viral shedding for at least 48 hours, in particular situations (ie, noncoughing family members than cannot quarantine from each other) repeatedly washing mouth and nose with H_2_O_2_ and maintaining clean surfaces with hypertonic solution may lower the circulating viral burden and protect the most fragile subjects.

## CONFLICT OF INTERESTS

The authors declare that there are no conflict of interests.

## AUTHOR CONTRIBUTIONS

Amedeo F. Capetti planned and designed the study and wrote the manuscript. Fabio Borgonovo and Valentina Morena contacted family physicians, performed swabs, and taught procedures. Gianfranco Dedivitiis and Angelica Lupo visited the patients and collected the data. Maria Vittoria Cossu and Matteo Passerini prepared bureaucracy for the EC and kept contacts with it. Giuliano Rizzardini found physical spaces, funded, and supervised the study.

## References

[1] Gandhi M , Yokoe DS , Havlir DV . Asymptomatic transmission, the Achilles' heel of current strategies to control covid‐19. N Engl J Med. 2020;382(22):2158‐2160. 10.1056/NEJMe2009758 32329972 PMC7200054

[2] Zhao J , Yuan Q , Wang H , et al. Antibody responses to SARS‐CoV‐2 in patients of novel coronavirus disease 2019 [published online ahead of print March 28, 2020]. Clin Infect Dis. 10.1093/cid/ciaa344 PMC7184337 32221519

[3] Furukawa NW , Brooks JT , Sobel J . Evidence supporting transmission of severe acute respiratory syndrome coronavirus 2 while presymptomatic or asymptomatic. Emerg Infect Dis. 2020;26(7):e201595 10.3201/eid2607.201595 PMC7323549 32364890

[4] Gao M , Yang L , Chen X . A study on infectivity of asymptomatic SARS‐CoV‐2 carriers. Respir Med. 2020;169:106026 10.1016/j.rmed.2020.106026 32513410 PMC7219423

[5] Cariani L , Orena BS , Ambrogi F , et al. Time length of negativization and cycle threshold values in 182 healthcare workers with Covid‐19 in Milan, Italy: an observational cohort study. Int J Environ Res Public Health. 2020;17(15):E5313 10.3390/ijerph17155313 32718008 PMC7432921

[6] Interim Guidance. Centers for disease control and prevention discontinuation of transmission‐based precautions and disposition of patients with COVID‐19 in healthcare settings. https://www.cdc.gov/coronavirus/2019-ncov/hcp/disposition-hospitalized-patients.html. Accessed 29 July 2020.

[7] WHO: Criteria for releasing COVID‐19 patients from isolation. https://www.who.int/news-room/commentaries/detail/criteria-for-releasing-covid-19-patients-from-isolation. Accessed 29 July 2020. 10.1016/j.phrs.2020.105063 PMC7834449 32663611

[8] Protocol G1.2020.0010603, 06/03/2020. Indirizzi in merito all'isolamento domiciliare. https://www.ats-milano.it/portale/Portals/0/emergenza%20coronavirus/Farmacia/nota%20ulteriore%20proorga%20PT%20AIFA%20fino%20al%2031%20agosto.pdf. Accessed 29 July 2020.

[9] Decontamination and Reuse of Filtering Facepiece Respirators. https://www.cdc.gov/coronavirus/2019-ncov/hcp/ppe-strategy/decontamination-reuse-respirators.html. Accessed 29 July 2020.

[10] Gruppo di lavoro ISS Biocidi COVID‐19: Raccomandazioni ad interim sui disinfettanti nell'attuale emergenza COVID‐19: presidi medico chirurgici e biocidi. https://www.epicentro.iss.it/coronavirus/pdf/rapporto-covid-19-19-2020.pdf Rapporto ISS COVID‐19. no. 19/2020. Accessed 29 July 2020.

[11] Medical Management Guidelines for Hydrogen Peroxide. www.atsdr.cdc.gov/MMG/MMG.asp?id=304%26tid=55#. Accessed 29 July 2020.

[12] Hofer T , Badouard C , Bajak E , Ravanat JL , Mattsson Å , Cotgreave IA . Hydrogen peroxide causes greater oxidation in cellular RNA than in DNA. Biol Chem. 2005;386:333‐337. 10.1515/BC.2005.040 15899695

[13] Caruso AA , Del Prete A , Lazzarino AI , Capaldi R , Grumetto L . Might hydrogen peroxide reduce the hospitalization rate and complications of SARS‐CoV‐2 infection? [published online ahead of print April 22, 2020] Infect Control Hosp Epidemiol. 2020:1‐2. 10.1017/ice.2020.170 PMC7308628 32319881

[14] Interim guidelines for collecting, handling, and testing clinical specimens from persons for coronavirus disease 2019 (COVID‐19). Updated 8 July 2020. https://www.cdc.gov/coronavirus/2019-ncov/lab/guidelines-clinical-specimens.html. Accessed 29 July 2020.

[15] Marty FM , Chen K , Verrill KA . How to obtain a nasopharyngeal swab specimen. N Engl J Med. 2020;382:e76 10.1056/NEJMvcm2010260 32302471

[16] Di Berardino F , Zanetti D , D'Amato G . Nasal rinsing with an atomized spray improves mucociliary clearance and clinical symptoms during peak grass pollen season. Am J Rhinol Allergy. 2017;31(1):40‐43. 10.2500/ajra.2016.30.4383 28234152

[17] Duration of Isolation and Precautions for Adults with COVID‐19. Updated July 22, 2020. https://www.cdc.gov/coronavirus/2019-ncov/hcp/duration-isolation.html. Accessed 29 July 2020.

[18] Winichakoon P , Chaiwarith R , Liwsrisakun C , et al. Negative nasopharyngeal and oropharyngeal swabs do not rule out COVID‐19. J Clin Microbiol. 2020;58‐20. 5e00297 10.1128/JCM.00297-20 32102856 PMC7180262

